# Assessment of Heart Rate Variability Thresholds from Incremental Treadmill Tests in Five Cross-Country Skiing Techniques

**DOI:** 10.1371/journal.pone.0145875

**Published:** 2016-01-04

**Authors:** Ibai Mendia-Iztueta, Kristen Monahan, Heikki Kyröläinen, Esa Hynynen

**Affiliations:** 1 Department of Biology of Physical Activity, University of Jyväskylä, Jyväskylä, Finland; 2 KIHU—Research Institute for Olympic Sports, Jyväskylä, Finland; Norwegian University of Science and Technology, NORWAY

## Abstract

The assessment of heart rate variability (HRV) thresholds (HRVTs) as an alternative of Ventilatory thresholds (VTs) is a relatively new approach with increasing popularity which has not been conducted in cross-country (XC) skiing yet. The main purpose of the present study was to assess HRVTs in the five main XC skiing-related techniques, double poling (DP), diagonal striding (DS), Nordic walking (NW), V1 skating (V1), and V2 skating (V2).Ten competitive skiers completed these incremental treadmill tests until exhaustion with a minimum of one to two recovery days in between each test. Ventilatory gases, HRV and poling frequencies were measured. The first HRV threshold (HRVT_1_) was assessed using two time-domain analysis methods, and the second HRV threshold (HRVT_2_) was assessed using two non-time varying frequency-domain analysis methods. HRVT_1_ was assessed by plotting the mean successive difference (MSD) and standard deviation (SD) of normalized R-R intervals to workload. HRVT_1_ was assessed by plotting high frequency power (HFP) and the HFP relative to respiratory sinus arrhythmia (HFP_RSA_) with workload. HRVTs were named after their methods (HRVT_1-SD_; HRVT_1-MSD_; HRVT_2-HFP_; HRVT_2-HFP-RSA_). The results showed that the only cases where the proposed HRVTs were good assessors of VTs were the HRVT_1-SD_ of the DS test, the HRVT_1-MSD_ of the DS and V2 tests, and the HRVT_2-HFP-RSA_ of the NW test. The lack of a wider success of the assessment of HRVTs was reasoned to be mostly due to the high entrainment between the breathing and poling frequencies. As secondary finding, a novel Cardiolocomotor coupling mode was observed in the NW test. This new Cardiolocoomtor coupling mode corresponded to the whole bilateral poling cycle instead of corresponding to each poling action as it was reported to the date by the existing literature.

## Introduction

Within the last three decades, blood lactate curves and gas exchange measurements from incremental exercise tests have been used for the assessment of endurance capacity and training zones, where two submaximal thresholds have been revealed [1]. However, the assessment of the two submaximal thresholds, being Ventilatory thresholds (VTs) or lactate thresholds (LTs), presents limitations such as laboratory dependence, high costs or an invasive nature [2].

At the beginning of the 21^st^ century, attempts to overcome these limitations began with the development of a new methodology for the assessment of submaximal thresholds based on Heart Rate Variability (HRV). The assessment of these thresholds, often referred to as HRV thresholds (HRVTs), is based on the links that HRV presents with the autonomous nervous system and respiratory sinus arrhythmia (RSA) [3–6].

The assessment of the first HRVT (HRVT_1_) has been conducted with complex time varying frequency analysis methods [7–10] but also with simple time analysis methods [2, 11]. The later ones are based on the virtual disappearance of the N-N (i.e. normalized R-R) interval variations, as seen in the trends of mean successive difference (MSD) and standard deviation (SD) of N-N, which represents vagal withdrawal.

The assessment of the second HRVT (HRVT_2_) has been conducted with time varying [8–10, 12–14] and non-time varying [13, 15] frequency analysis methods. The frequency analysis of HRV decomposes the N-N time-dependent signal into its sinusoidal components, obtaining the so-called power spectral density (PSD). This PSD is then under-divided into low and high frequency (HF) bands. The HF band results from breathing (respiratory sinus arrhythmia or RSA) and mainly vagal cardiovascular control, but it is also related to Cardiolocomotor coupling (LOC) when summit to exercises where the upper body is involved [6, 13, 15]. The latest studies indicate that the most sensitive methods for HRVT_2_ assessment in exercises where the upper body strikes contribute to propulsion are time varying methods that discard the power in the HF band (HFP) relative to the LOC (HFP_LOC_) to solely use the HFP relative to the RSA (HFP_RSA_) [13, 15].

To the best of our knowledge, the assessment of HRVTs has been conducted in varied exercise modes, including exercises using upper body movements, such as front crawl swimming [15] and ski mountaineering [13, 14], but never in cross-country (XC) skiing. A characteristic of XC skiing is that, like swimming, it consists of different techniques with different relationships between breathing frequency (BF) and poling frequency (PF) [16, 17], which would presumably imply different peak patterns in the HF band. The five main techniques used by XC skiers are Diagonal Striding (DS), Nordic Walking (NW), Double Poling (DP), V1 skating (V1) and V2 skating (V2). All techniques seem to share a tendency for an increase in BF-PF coupling with increasing workload intensities. Moreover, it seems that the PF-BF coupling is the weakest in the DS and NW techniques and strongest in the DP technique [13, 16–20].

The main aim of the present study was to assess HRVTs in the five main techniques used by cross country skiers, and the secondary aim was to evaluate how the Cardiolocomotor interactions affected these assessments. For these purposes, five discontinuous incremental treadmill tests until exhaustion were conducted. Two time analysis methods were selected for the assessment of HRVT_1_. The HRVT_1_ obtained from MSD was named HRVT_1-MSD_, and the HRVT_1_ obtained from SD was named HRVT_1-SD_. For the assessment of HRVT_2_ two non-time varying frequency analysis methods were used. The HRVT_2_ obtained from HFP was named HRVT_2-HFP_, and the HRVT_2_ obtained from HFP_RSA_ was named HRVT_2-HFP-RSA_.

## Methods

A group of competitive XC skiers performed five incremental tests until exhaustion in a time frame of two to three weeks with at least one day between tests. The tests were performed in randomized order.

### Subjects

Ten competitive national level XC skiers (5 male and 5 female), with ages ranging from 19 to 30 years participated in the study (Table 1). All athletes were healthy and had just completed their XC ski race season. All subjects gave written informed consent agreeing to the terms and conditions of the study, and all possible risks and benefits were explained. The study was approved by the Ethics Committee of the University of Jyväskylä. The amount of subcutaneous fat was estimated from a four site skinfold measurement (m. biceps, m. triceps, os. scapula and, os. crista iliaca) [21].

**Table 1:** Characteristics of the study subjects (n = 10).

Characteristics	Mean ± SD	
	Males	Females
**Age (years)**	23.4 ± 4.5	26.0 ± 3.9
**Height (cm)**	180.2 ± 3.1	166.6 ± 4.6
**Body mass (kg)**	72.2 ± 2.7	60.2 ± 4.1
**Fat (%)**	10.3 ± 1.5	19.4 ± 2.9
$\overset{{}^{\circ}}{V}$**O**_**2**_**max (ml/kg/min)**	73.2 ± 1.1	59.9 ± 3.5

### Procedure

#### Incremental Tests

NW, DP, DS, V1 skating, and V2 skating were the techniques used for the five incremental tests. All tests were performed on a large motor-driven treadmill (Rodby RL3500E, Rodby Innovations, Vänge, Sweden) located in an indoor laboratory setting. All participants wore a harness that was attached to a rope which hung from a metallic frame in the ceiling above the treadmill for safety. Marwe Classic 800 C and Marwe Skate 610 A roller skis (Hyvinkään Kumi, Hyvinkää, Finland) were used. One Way ski poles (One Way Sport, Vantaa, Finland) were equipped with special rubber tips designed to ensure an optimal grip while roller skiing on the motor-driven treadmill (Biomekanikk AS, Oslo, Norway). Before each incremental test, the subjects performed a 10 to 15 minute warm up with the same technique they were performing in the test on that day. In all protocols, workload increased every three minutes. At the end of every stage, the treadmill was stopped for 10 to 15 seconds for blood lactate samples (not used in this study) from the standing subjects. This small timeframe was included in the three minute stage. The highest 20 second mean values obtained during the tests were defined as peak values. The highest 20 second $\overset{{}^{\circ}}{\text{V}}$O_2_ mean value ($\overset{{}^{\circ}}{\text{V}}$O_2_peak) among the five tests was defined as the $\overset{{}^{\circ}}{\text{V}}$O_2_max value for the person if two or more of the following criteria were met: a HR within 10 beats of the age predicted maximal HR (220 –age); a respiratory exchange ratio superior to 1.1; or a plateau in $\overset{{}^{\circ}}{\text{V}}$O_2_ with increasing workload [13]. The PF was measured with a chronometer by timing ten poling cycles in the last minute of each stage.

In the DP and the V2 protocols inclination was maintained constant at 3% and 5%, respectively, whereas speed increased 2 km/h with every stage from the initial speeds of 8 km/h for men and 7 km/h for women. In the DS and V1 tests, speed was maintained constant at 10 km/h for men and 9 km/h for women, whereas inclination increased 2% with every stage from the initial 3% inclination. The NW tests followed a protocol that has been used for more than 30 years in Finland (Table 2) [22].

**Table 2:** Nordic Walking test protocol, women started from stage 1 and men from stage 2.

Stage	1	2	3	4	5	6	7	8	9	10
**Inclination (%)**	4	7	9	12	14	17	20	22	23	25
**Speed (km/h)**	6.0	6.0	6.6	6.6	7.0	7.0	7.0	7.2	7.6	7.8

#### Assessment of First and Second Ventilatory Thresholds

A portable Oxycon Mobile spiroergometer (Viasys Healthcare GmbH, Hoechberg, Germany) was used for the gas measurements. The spiroergometer’s main sampling unit was lightweight and attached to the upper back of the participants. Gas and volume calibrations were carried out twice before every test. The average breath-by-breath values of the last 90 seconds of every stage became the subject's values for the stage, a timeframe that complied with the interval lengths used for the HRV analysis [15]. VTs were determined by visual analysis of the breakpoints of different variables that were well documented in the literature [23]. For the determination of VT_1_, $\overset{{}^{\circ}}{\text{V}}$E-$\overset{{}^{\circ}}{\text{V}}$O_2_ and $\overset{{}^{\circ}}{\text{V}}$CO_2_-$\overset{{}^{\circ}}{\text{V}}$O_2_ were plotted, whereas the determination of VT_2_, was assessed based on the $\overset{{}^{\circ}}{\text{V}}$E-$\overset{{}^{\circ}}{\text{V}}$O_2_ and the $\overset{{}^{\circ}}{\text{V}}$E-$\overset{{}^{\circ}}{\text{V}}$CO_2_ graphs. In the instances where the referred graphs did not allow determination of VTs, the graphs that plotted Ventilatory Equivalent of Carbon Dioxide ($\overset{{}^{\circ}}{\text{V}}$E/$\overset{{}^{\circ}}{\text{V}}$CO_2_) and Ventilatory Equivalent of Oxygen ($\overset{{}^{\circ}}{\text{V}}$E/$\overset{{}^{\circ}}{\text{V}}$O_2_) with $\overset{{}^{\circ}}{\text{V}}$O_2_ were used.

#### Assessment of First and Second Heart Rate Variability Thresholds

A Suunto t6 (Suunto Oy, Vantaa, Finland) HR monitor with a sampling frequency of 1000 Hz for the recording of the R-R intervals was used throughout each test. From the HRV recordings, the R-R interval series relative to the last 90 seconds of each step were extracted, as HF oscillations during these periods were considered stationary [15]. The R-R interval series were then automatically filtered with the software Firstbeat Health 3.1.1.0 (Firstbeat Technologies Oy, Jyväskylä, Finland) for the correction of the eventual artefacts and ectopic beats, which has been proven to be fast, accurate and reliable [24]. The normalized R-R (also named as N-N) time series, were then analysed by the software Kubios HRV 2.1 (Biosignal Analysis and Medical Imaging Group BSAMIG, Kuopio, Finland). HRVT_1_ was assessed from two time-domain HRV analyses methods [11]. MSD and the SD were graphically plotted against workload. HRVT_1-SD_ and HRVT_1-MSD_ were set by visual interpretation at the point where there was no further decrease in the trends of these two parameters [11].

HRVT_2_ was assessed with two time-domain HRV analyses methods [15]. The HFP and the HFP_RSA_ were plotted against workload. One and sometimes two increases were noticed in the trends of both variables. HRVT_2-HFP_ and HRVT_2-HFP-RSA_ were set by visual interpretation at the point from which the last increase started [15].

The N-N series were first converted into an equidistantly sampled form, and the HR trend and part of the LF oscillations were then removed [15]. The PSD was estimated by an autoregressive model of order 12 or 18, depending on the type of test. An order 12 was applied to the DS and NW tests. However, an autoregressive model order 18 was considered to be more appropriate for the DP, V1, and V2 tests, because unlike the previously studied disciplines [13–15], these three disciplines use symmetric bilateral upper body strikes. The LF band was set between 0.04 and 0.15 Hz, and the HF band was set between 0.15 and 2.00 Hz. HFP was computed as the spectral power in the referred HF range, whereas HFP_RSA_ was computed as the power from frequencies ranging from 0.04 Hz to a varying cut-off frequency which corresponded to the borderline between the RSA-related and LOC-related HF components [13, 15]. The frequencies of the PSD peaks on HFP_RSA_ and HFP_LOC_ were named as fHF and pfHF, respectively. In the occurrence where HFP_RSA_ and HFP_LOC_ could not be divided, the frequency of the density peak was considered to be both fHF and pfHF. To verify that the HFP_RSA_ and HFP_LOC_ components were indeed related to respiration and locomotion, all of the spectrums (n = 321: 45 maximal tests with 5–10 stages each) were visually inspected and fHF and pfHF were compared to the corresponding BF and PF measured by spiroergometer, and chronometer.

The differentiation of the two HF band components in the five different tests occurred as follows. In the HF ranges of spectrums in the V2 tests, two clear PSD peaks emerged, the first peak corresponding to RSA and the second peak corresponding to LOC. In the DS tests, the main peak corresponded to RSA and the last and often non prominent peak's frequency (pfHF) corresponded to the double of PF. In the spectrums of the NW tests, two main peaks emerged. The first peak corresponded to RSA, and the second peak to PF. Lastly, in the vast majority of spectrums obtained from the V1 and DP tests, a single peak emerged in the HF band, preventing to differentiate two HF components. Therefore the decision was made to not split HFP in any of the spectrums of the V1 and DP tests, and the frequencies of their main peaks were decided to be both fHF and pfHF.

### Statistical Analysis

A between methods agreement was used to evaluate whether there was an agreement or bias between the variables (i.e. VTs, BF and PF) determined from the reference methods (i.e. timer for PF and Ventilatory gases for the rest of variables) and the corresponding variables assessed from the alternative HRV-related methods (i.e. HRVTs, fHF and pfHF). The analyses included (a) an evaluation of the relationships between parameters using Pearson’s r correlation coefficients and linear regression lines, (b) an examination of the level of agreement using 95% limits of agreement according to Bland-Altman, and (c) a comparison of mean values using paired t tests. The agreement analysis between VTs and HRVTs was conducted with HR values because of its practical applicability. All the data are reported as the mean ± SD and the statistical significance was set at p ≤ 0.05 for all tests. All statistical analyses were performed using IBM SPSS Statistics 20 software (SPSS Inc, Chicago, IL, USA). The normal distribution of the data was verified by One-Sample Kolmogorov-Smirnov Test and the magnitude of the correlations was assessed according to Hopkins' scale [25].

## Results

In every subject, the test reaching the highest $\overset{{}^{\circ}}{\text{V}}$O_2_peak value met the $\overset{{}^{\circ}}{\text{V}}$O_2_max criteria, meaning that all subjects reached their maximal aerobic capacity in at least one of the five tests. Seven subjects reached the highest $\overset{{}^{\circ}}{\text{V}}$O_2_peak value in the DS test and the remaining 3 subjects reached their highest $\overset{{}^{\circ}}{\text{V}}$O_2_peak value in the NW test. Males' $\overset{{}^{\circ}}{\text{V}}$O_2_max was 73.2 ± 1.1 ml/kg/min, whereas females' value was 59.9 ± 3.5 ml/kg/min.

### Agreements between Ventilatory and Heart Rate Variability Thresholds

All VTs except the VT_1_ of one subject's DP test were assessed. The R-R interval data from five maximal tests (2 DP tests, 2 V1 tests and 1 V2 test) were excluded due to excess of artefacts, and hence, none of their HRVTs could be assessed. Moreover, HRVT_1-MSD_ could not be assessed in one of the DS tests because of the abnormal behaviour of its trend. Additionally, the HRVT_2-HFP-RSA_ was not assessed in the DP and V1 tests because it was not possible to divide the HFP spectrums into two components. Besides the aforementioned cases where HRVTs could not be assessed, the reminders of HRVTs were assessed, including the HRVT_1_ corresponding to the VT_1_ that could not be assessed by the Ventilatory gas exchange method. A summary of the agreement between VTs and HRVTs can be seen in Table 3.

**Table 3:** Mean (± SD) differences between the first (A & B) and second (C & D) thresholds determined by HRV and conventionally (spiroergometry).

A) VT_1_-HRVT_1-SD_	Agreement (bpm)	P	R	P	95% limits of agreement (bpm)
**Double poling**	9 ± 21	0.281	-0.03	0.955	-32 / 51
**Diagonal striding**	-1 ± 8	0.746	0.77	0.009	-17 / 15
**Nordic walking**	9 ± 8	0.007	0.68	0.031	-4 / 22
**V1 skating**	-2 ± 13	0.619	-0.28	0.495	-27 / 23
**V2 skating**	6 ± 10	0.112	0.26	0.492	-14 / 26
**B) VT**_**1**_**-HRVT**_**1-MSD**_					
**Double poling**	-9 ± 9	0.039	0.88	0.009	-27 / 9
**Diagonal striding**	-6 ± 8	0.070	0.75	0.020	-21 / 10
**Nordic walking**	-9 ± 16	0.277	0.24	0.502	-37 / 25
**V1 skating**	-9 ± 11	0.049	0.19	0.654	-31 / 12
**V2 skating**	-3 ± 8	0.364	0.77	0.016	-18 / 13
**C) VT**_**2**_**-HRVT**_**2-HFP**_					
**Double poling**	-8 ± 19	0.250	0.54	0.172	-45 / 28
**Diagonal striding**	11 ± 13	0.027	0.80	0.006	-36 / 15
**Nordic walking**	-4 ± 9	0.180	0.44	0.207	-23 / 14
**V1 skating**	-18 ± 10	0.001	-0.28	0.510	-37 / 1
**V2 skating**	-8 ± 8	0.019	0.81	0.008	-24 / 8
**D) VT**_**2**_**-HRVT**_**2-HFP-RSA**_					
**Diagonal striding**	-9 ± 13	0.055	0.66	0.036	-35 / 17
**Nordic walking**	-1 ± 7	0.818	0.82	0.026	-15 / 14
**V2 skating**	-6 ± 8	0.064	0.37	0.334	-21 / 10

Mean differences between the first threshold assessed conventionally (VT_1_) and A) estimated by the Standard Deviation method of HRV (HRVT_1-SD_) and B) estimated by the Mean Successive Difference method of HRV (HRVT_1-MSD_). Mean differences between the second threshold assessed conventionally (VT_2_) and A) estimated by the High Frequency Power method of HRV (HRVT_2-HFP_) and B) estimated by the Respiratory Sinus Arrhythmia related component of High Frequency Power (HRVT_2-HFP-RSA_). HRV = heart rate variability; r = correlation coefficient; p = statistical significance (p ≤ 0.05).

#### First Threshold

**Fig 1:**
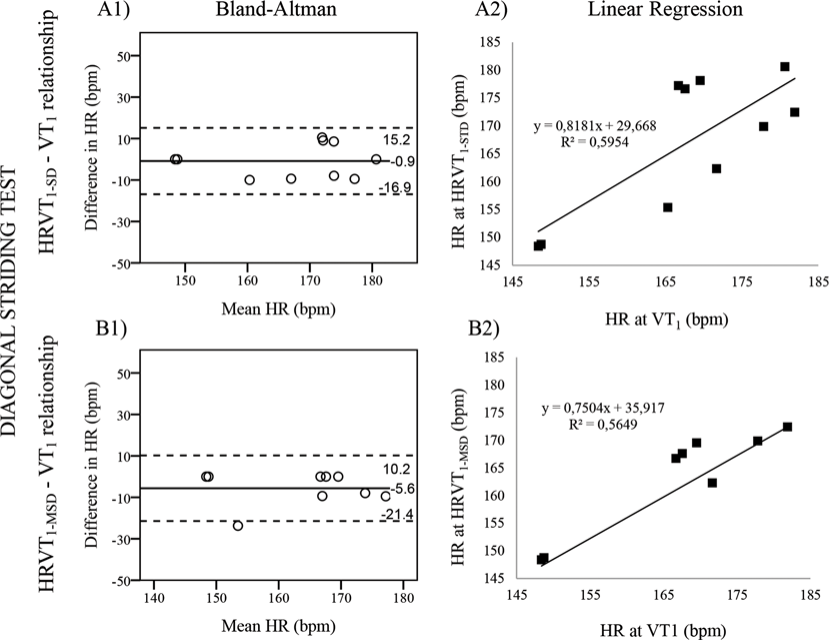
Validity testing of the HRVT_1-SD_ (A1, A2) and HRVT_1-MSD_ (B1, B2) for the assessment of VT_1_ during diagonal striding test. Bland-Altman (A1 & B1) plots the respective difference in heart rate (HR) between VT_1_-HRVT_1-SD_ and VT_1_-HRVT_1-MSD_ for each individual against their respective means. Dashed lines represent the limits of agreement corresponding to ± 1.96 SD. The best fitting linear regression lines (A2 & B2) are also displayed together with their equations, enabling a prediction of future HR values. HRVT_1-SD_ & HRVT_1-MSD_ = heart rate variability thresholds as determined from standard deviation and from mean successive difference of normalized R-R intervals, respectively; VT_1_ = first Ventilatory threshold.

With regards to the SD method, the NW test was the only test that showed statistical difference between HRVT_1-SD_ and VT_1_ (p = 0.007). These variables where significantly correlated only in the DS (r = 0.77, p = 0.009) and NW (r = 0.68, p = 0.031) tests. The biases show that HRVT_1-SD_ slightly underestimated VT_1_ in the DS and V1 tests (-1 and -2 bpm, respectively), but VT_1_ was overestimated in the other tests. The 95% limits of agreement were narrowest in NW and DS (-4/20 and -17/15 bpm, respectively), and was widest in DP (-32/51 bpm). Based on the regression lines, HRVT_1-SD_ explained 0.1% of the total variability of VT_1_ in DP, 59.5% in DS, 46.1% in NW, 8.1% in V1 and 7.0% in V2. Therefore, it seems like the agreement between HRVT_1-SD_ and VT_1_ was best in the DS test (Fig 1A) since their means were not significantly different, the bias was very small, they were strongly correlated and the regression line explained a good amount of the total variability of VT_1_.

With regards to the MSD method, the DP and V1 tests were the only tests that showed statistical differences between HRVT_1-MSD_ and VT_1_ (p = 0.039 and 0.049, respectively). These variables where significantly correlated in the DP (r = 0.88, p = 0.009), V2 (r = 0.77, p = 0.016) and DS (r = 0.75, p = 0.020) tests. All the biases showed a certain underestimation of HRVT_1-MSD_ over VT_1_; the biases where smallest in V2 and DS (-3 and -6 bpm, respectively). The limits of agreement were also narrowest in the V2 and DS tests (-18/13 and -21/10 bpm, respectively). Based on the regression lines, HRVT_1-MSD_ explained 77.6% of the total variability of VT_1_ in DP, 56.5% in DS, 5.8% in NW, 3.6% in V1 and 58.5% in V2. Therefore, it seems like the agreement between HRVT_1-MSD_ and VT_1_ was best in the DS (Fig 1B) and V2 tests, since in both tests the variables were not significantly different, were strongly correlated and the agreement intervals were not that large. The equation of the best fitting linear regression line for the V2 test was y = 1.1007x - 19.305.

#### Second Threshold

**Fig 2:**
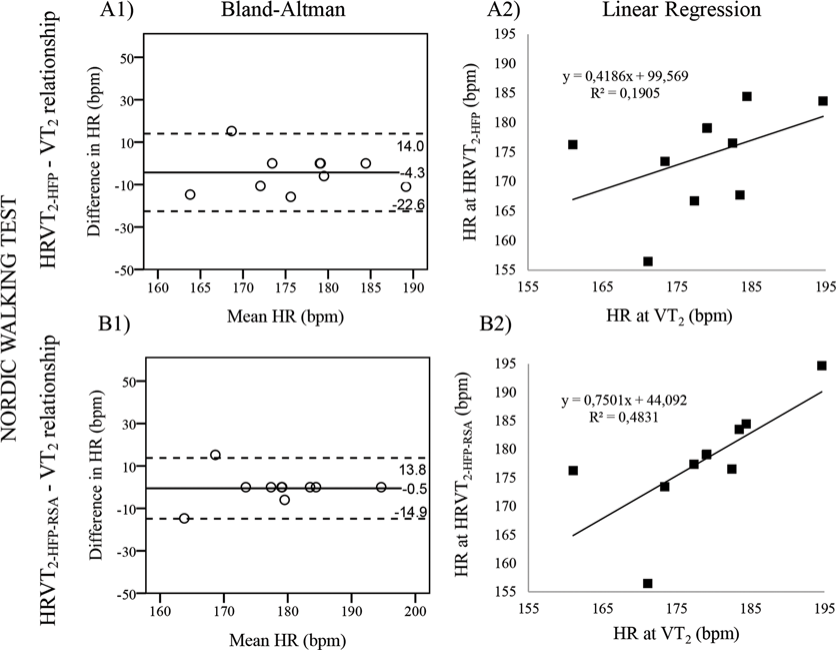
Validity testing of the HRVT_2-HFP_ (A1, A2) and HRVT_2-HFP-RSA_ (B1, B2) for the assessment of VT_2_ during Nordic walking test. Bland-Altman (A1 & B1) plots the respective difference in Heart Rate (HR) between VT_2_-HRVT_2-HFP_ and VT_2_-HRVT_2-HFP-RSA_ for each individual against their respective means. Dashed lines represent the limits of agreement corresponding to ± 1.96 SD. The best fitting Linear Regression lines (A2 & B2) are also displayed together with their equations, enabling a prediction of future HR values. HRVT_2-HFP_& HRVT_2-HFP-RSA_ = heart rate variability thresholds as determined from the high frequency power and from the high frequency power component related to respiratory sinus arrhythmia, respectively; VT_2_ = second Ventilatory threshold.

With regards to the HFP method, the DS, V1 and V2 tests showed statistical differences between HRVT_2-HFP_ and VT_2_ (p = 0.027, 0.001 and 0.019, respectively). These variables where significantly correlated only in the DS (r = 0.80, p = 0.006) and V2 (r = 0.81, p = 0.008) tests. The smallest bias was found in NW (-4 bpm) and the biggest biases were found in V1 and DS (-18 and 11 bpm, respectively). The 95% limits of agreement were narrowest in V2, NW and V1 (-24/8, -23/14 and -37/1 bpm, respectively), and was widest in DP (-45/28 bpm). Based on the regression lines, HRVT_2-HFP_ explained 28.6% of the total variability of VT_1_ in DP, 63.1% in DS, 19.1% in NW, 7.5% in V1 and 65.5% in V2. Therefore it seems that in none of the tests HRVT_2-HFP_ was a particularly good assessor of VT_2_. The lack of agreement between HRVT_2-HFP_ and VT_2_ of the NW test is well seen in Fig 2A.

With regards to the RSA method, none of the three tests showed statistical differences (p ≥ 0.05) between HRVT_2-HFP-RSA_ and VT_2_. These variables were significantly correlated only in the NW (r = 0.82, p = 0.026) and DS (r = 0.66, p = 0.036) tests. The biases of all three tests were negative, representing an underestimation of HRVT_2-HFP-RSA_ over VT_2_. The smallest bias was found in NW (-1 bpm) and the biggest in DS (-9 bpm). The limits of agreement was widest in DS (-35/17 bpm) and based on the regression lines, HRVT_2-HFP-RSA_ explained 44.1% of the total variability of VT_2_ in DS, 48.3% in NW and 13.3% in V2. Therefore, it seems like the agreement between HRVT_2-HFP-RSA_ and VT_2_ was close to optimal in the NW test (Fig 2B), since apart from being very strongly correlated and presenting no significant difference, the bias was very small, the agreement interval relatively small, and the regression line explained a good level of the total variability of VT_2_.

### fHF-BF and pfHF-PF Agreements

For all tests except for the V2 test, there was no significant difference between the mean of fHF and the mean of the timed BF. These variables were very strongly correlated (r ≥ 0.8) in DS, NW and V2, and strongly correlated (r = 0.6–0.8) in DP and V1 (Table 4A). In regards of the agreement between pfHF and the timed PF, the variables were significantly different and uncorrelated (p ≥ 0.05) in the DS and V1 tests. Other than this, there were no significant differences in the rest of the tests and the level of correlations were either very strong (NW and V2) or strong (DP) (Table 4B).

**Table 4:** Mean (± SD) differences between Breathing (A) and Poling (B) Frequencies determined by HRV and conventionally.

A) fHF-BF	Agreement (breaths/min)	P	R	P	95% limits of agreement (breaths/min)
**Double poling**	1 ± 3	0.445	0.72	0.045	-5 / 6
**Diagonal striding**	0 ± 2	0.801	0.87	0.001	-4 / 4
**Nordic walking**	0 ± 1	0.976	0.97	0.000	-3 / 3
**V1 skating**	-1 ± 2	0.136	0.73	0.041	-5 / 3
**V2 skating**	2 ± 2	0.016	0.91	0.001	-3 / 6
**B) pfHF-PF**	**(cycles/min)**				**(cycles/min)**
**Double poling**	1 ± 2	0.136	0.77	0.024	-3 / 6
**Diagonal striding**	2 ± 2	0.021	0.42	0.222	-2 / 6
**Nordic walking**	1 ± 1	0.116	0.96	0.000	-2 / 3
**V1 skating**	2 ± 2	0.029	0.54	0.166	-2 / 5
**V2 skating**	1 ± 1	0.086	0.93	0.000	-2 / 4

A) Mean differences between breathing frequency measured by respiratory measurements (BF) and estimated by HRV analysis (fHF). B) Mean differences between poling frequencies measured by timed measurements (PF) and estimated by HRV analysis (pfHF). HRV = heart rate variability; r = correlation coefficient; p = statistical significance (p ≤ 0.05).

### Relationships between Breathing and Poling Frequencies

**Fig 3:**
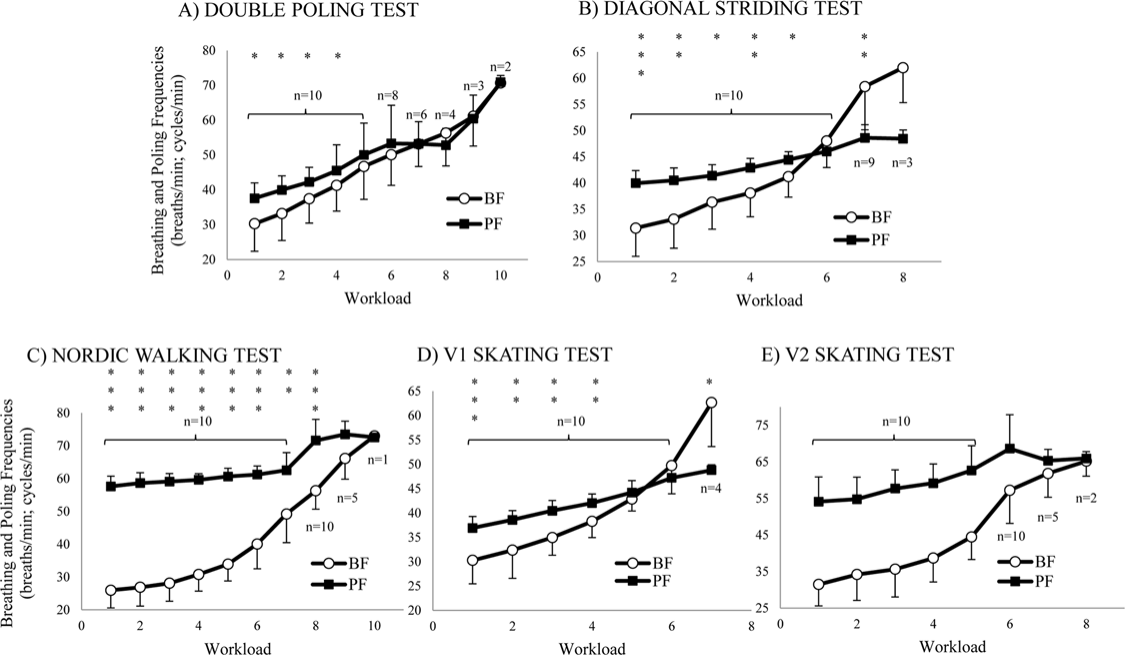
Evolution of Poling and Breathing Frequencies. Mean (± SD) differences of poling frequency (PF) and breathing frequency (BF) during the five different incremental tests. * = significant difference between PFs and BFs, * = p ≤ 0.05; ** = p ≤ 0.01; *** = p ≤ 0.001.

In the five tests, there was a tendency for an increased coupling between the BF and PF as workload increased and there was always a higher PF than BF during the initial workloads. In the DP test, PF and BF seemed to be quite similar; PF was only significantly higher than BF in the first four stages. However, in the V1 and DS tests the BF trend significantly surpassed the PF trend in a cross-like pattern; significant differences between BF and PF where observed in the initial and last stages. With regards to the NW and V2 tests, substantially higher BF than PF values were presented in all except the last two stages (Fig 3).

## Discussion

The present study, the first in assessing HRVTs in XC skiing-related techniques, shows results supporting that the assessment of HRVTs in XC skiing is a difficult task due to the strong involvement of upper body movements. More precisely, the VT_1_ was only successfully estimated by the HRVT_1-SD_ and by the HRVT_1-MSD_ in the DS test, and by the HRVT_1-MSD_ in the V2 test. The only good assessor of VT_2_ was the HRVT_2-HFP-RSA_ of the NW test. These assessments support our hypothesis that the VTs would be better replaced by HRVTs in techniques where the involvement of the upper body is moderate (DS and NW) and would by worse replaced when the upper body involvement is highest (DP). Furthermore, the frequency analysis of HRV used in the present study gives an interesting view on how the different poling actions of the studied XC skiing related techniques modulate the HR in different ways, among which a previously undocumented mode of Cardiolocomotor modulation was observed. This new mode of Cardiolocomotor modulation was observed in the power spectrums of the HF bands of the NW test, and was related to half of the rate of arm movements.

### Assessment of Ventilatory and Heart Rate Variability Thresholds

Despite the determination of both VT_1_ and VT_2_ being challenging because the plots did not always clearly show two inflection points, all VTs except the VT_1_ of one subject's DP test were determined. These difficulties were expected as a result of a previous V2 study [18] which concluded that the determination of VT_2_ from Ventilatory gas analysis must be used cautiously in exercises involving upper body movements. It was explained that the PF entrains the BF, preventing the usual inflection-like behaviour of the Ventilatory variables.

With regards to the assessment of HRVTs, taking into account that we hypothesized difficulties for obtaining HRVT_2-HFP-RSA_ in the DP test, it was not a surprise that HRVT_2-HFP-RSA_ could not be assessed in DP and V1. This occurred because the strong synchronisation between PF and BF provoked the appearance of single merged peaks in the HF band. However, the reminders of HRVTs were assessed, including the VT_1_ which could not be assessed from the ventilatory method.

The HFP_RSA_ and HFP_LOC_ patterns of the studied tests, disregarding the NW test, were not unexpected as they closely corresponded to the frequencies of BF and PF. The appearance of HFP_LOC_ corresponded to the rate of arm movements, which was the same as the PF in V2, V1 and DP, all using the bilateral synchronous polling pattern. In the DS test, HFP_LOC_ also corresponded to the rate of arm movements, but in this particular case it was the same as the double of PF due to DS using a bilateral asynchronous poling (i.e. each poling cycle consists of two poling actions, one for each hand), which was reported in front crawl swimming [15] and ski mountaineering [13]. The same HFP_LOC_—PF relationship presented in DS was expected to be observed in the NW test, however, this was not the case. The frequency of HFP_LOC_ corresponded to PF, or in other words, to half of the rate of arm movements. As a result the HFP_LOC_ corresponds to the whole bilateral poling cycle instead of each poling action. Thus, during these four techniques using roller skies, the pfHF closely corresponded to the rate of arm movements (i.e 2 PF in DS and PF in V2, V1 and DP), whereas in NW pfHF corresponded to half of the rate of arm movements, “ignoring” the action of half of the poling actions. The correspondence of HFP_LOC_ to half of the rate of arm movements, to the best of our knowledge, is unreported, and it could be considered to be a new Cardiolocomotor coupling mode.

We can speculate that the appearance of this novel LOC mode and the failure to find a peak at the double of PF must have been related, in one way or another, to the distinctive nature that NW is the only discipline that does not use roller skies. On one hand, the absence of a stride phase shortens the stride length, which demands a higher PF for maintaining a given workload. This implied that, in many spectrums (in 47 out of 87 spectrums) the rate of arm movements was higher than the upper frequency limit of PSD (2 Hz), which would explain the failure to find a peak at the rate of arm movements. On the other hand, the absence of a stride phase most likely also entails lower poling forces due to their propulsion-wise transcendance being reduced. These lower forces could somewhat explain the “missing” peaks in the HF band.

### Agreements between Ventilatory and Heart Rate Variability Thresholds

The agreement between VT_1_ and the two different equivalents assessed from time-domain analysis were not as good as what were found in a cycle-ergometer [11] and a walking study [2]. The only test where our HRVT_1-SD_ closely corresponded to VT_1_ was during the DS test, and HRVT_1-MSD_ was only a relatively good assessor of VT_1_ in the V2 and DS tests. In the DS test the means of HRVT_1-SD_ and VT_1_ were not significantly different, showed virtually no bias, and were strongly correlated (r = 0.77). However, previous studies [2, 11] reported stronger correlations (r = 0.95 and or r = 0.89, respectively). Moreover, the linear regression of our DS test only explained 59.5% of the total variability compared to the 89.6% explanation reported in the walking study [2]. In our V2 and DS tests, the means of HRVT_1-MSD_ and VT_1_ were not significantly different and were strongly correlated (r = 0.77 and 0.75, respectively). However, the cycle ergometer study [11] has again reported a stronger correlation (r = 0.89). Furthermore, the cycle ergometer study showed no bias between the means. On the contrary, the negative biases (-3 and -6 bpm for the V2 and DS tests, respectively) found in our study represented a slight underestimation of HRVT_1-MSD_ over VT_1_. In the reminder of the tests, both HRVT_1-SD_ and HRVT_1-MSD_ failed to successfully correspond to VT_1_.

It is remarkable that, in the cycle ergometer based study [11] where both HRVT_1-SD_ and HRVT_1-MSD_ were assessed, the two thresholds corresponded to the same workloads in every case, whereas this was not the case in the present study. Based in our study, it seems that the use of the HRVT_1-MSD_ is preferable to HRVT_1-SD_ in XC skiing since the former was a valid assessor of VT_1_ in two techniques (V2 and DS) whereas the latter was only a valid assessor in the DS technique.

With regards to HRVT_2_, the agreement between VT_2_ and different equivalents assessed from frequency-domain analysis usually did not correspond to the close agreements reported by a swimming study [15] and a ski mountaineering study [13]. Nevertheless, it is important to note that the swimming study [15] compared the HRVT_2_ to the second lactate threshold and not to the VT_2_. Our HRVT_2-HFP_ was not a proper assessor of the VT_2_ in either of the tests. However, it seems like HRVT_2-HFP-RSA_ was a good assessor of VT_2_ in the NW test and a fair assessor in the DS test, even though it was not a valid assessor in V2. In the NW test, the means of HRVT_2-HFP-RSA_ and the VT_2_ were not significantly different, they had virtually no bias and they were strongly correlated (r = 0.82). Moreover, the agreement interval was -15 to 14 bpm and the regression line explained 48.3% of the total variability. These results are similar to the results reported in the aforementioned studies [13, 15]. In these studies, correlations were also high (r = 0.63 and r = 0.93, respectively), the biases were low (-3 and -1 bpm, respectively), and the agreement intervals were quite narrow (-22 to 14 bpm and -7 to 5 bpm, respectively). In the DS test, the agreement between HRVT_2-HFP-RSA_ and VT_2_ was not as good as in the NW test or in the referred two studies, due to the HRVT_2-HFP-RSA_ of the DS test considerably underestimating VT_2_ (-9 bpm bias). Therefore, HRVT_2-HFP-RSA_ would probably not be a valid enough alternative of VT_2_ in the DS test regarding the applicability in the field, due to its underestimation exceeding the magnitude of the daily variations in HR. A previous study [26] supports this statement, as it concluded a change in submaximal HR of more than 3 bpm could be considered a meaningful change under controlled conditions. Finally, it is also important to note that in our results, similar to the aforementioned literature, when HRVT_2-HFP-RSA_ was determined, it was a better assessor of VT_2_ than HRVT_2-HFP_.

A V2 study [18] reported that the assessment of VT_2_ in exercises involving upper body movements needs to be used cautiously because there is a high entrainment between the BF and PF. With increasing intensity ventilation shows two inflection points during the regular assessment of VTs, when PF is an added variable, PF alters ventilation and we no longer can observe the inflection points. As a result, the rhythm of PF alters the usual Ventilatory response in which the assessment of VTs (and hence also the assessment of HRVTs) is based on. This could be one reason why the assessment of HRVTs in this study was not always successful. However, some studies [13–15] successfully assessed HRVT_2_ using the same methods in exercises using upper body locomotion. Finally, as apart from the nature of the exercises, the selected incremental treadmill protocols and the method used for the VTs’ assessment were the only differences found in these studies [13, 15], it is possible that the selected protocols and the methods used for the VTs’ assessment could be improved upon in the present study.

### Breathing and Poling Frequencies

With regards to fHF-BF and pfHF-PF agreements, BF and PF assessed by HRV (i.e fHF and pfHF) and their respective equivalents determined conventionally did not present any high variances. If any, the HRV measurements tended to slightly overestimate the reference measurements. This was expected from the literature [13, 15, 27], and thus, the study was successful in regards to this matter.

With regards to the relationships between breathing and poling frequencies, all five tests had a tendency to show an increased BF-PF coupling with increased workload and a higher PF than BF during the initial workloads. This is in line with the previous literature [18]. With regards to the five specific tests, three kinds of BF-PF relationships were observed: 1) In the DP test, BF and PF were synchronised (1:1 BF to PF ratio) almost from the very beginning; 2) the V1 and DS tests showed statistical differences in early and late stages as the BF trend surpassed the PF trend in a cross-like pattern; and 3) the NW and V2 tests presented substantially different BF and PF values except in the last two stages. All results agree with the literature [13, 16–20] except for the significantly different mean values observed in the final stages of the V1 and DS tests that lead to the cross-like pattern. This cross-like pattern phenomenon, to the best of our knowledge, was unreported to date. Moreover, our initial hypothesis stating that there would be an especially high BF to PF ratio for the DS test and a lower BF-PF entrainment for the DS and NW tests was confirmed. Nevertheless, apart from the DP test, the V1 test also showed a 1:1 BF to PF ratio predominance among the subjects, and in the V2 test, a high BF-PF correlation was also confirmed. In the V2 test, most of the subjects adopted 1:1 and/or 1:2 BF to PF ratios as reported in the previous literature [16, 18].

## Conclusion

The assessment of HRVTs is a field of research worthwhile to study as it aims to offer an economical, non-laboratory dependant and non-invasive alternative to the conventional Ventilatory and lactate thresholds. Nevertheless, this paper shows that the assessment of HRVTs, when applied to XC skiing-related techniques, is a difficult task that requires expertise due to the poling actions altering the usual Ventilatory response in which the assessment of HRVTs is based off of. Moreover, this paper also shows that the assessment of HRVTs in XC skiing requires the selection of an appropriate method, which might not always ensure the successful determination of HRVTs. The only cases where this study successfully assessed VT_1_ from HRV were the two time-domain HRV methods in the DS test and the MSD-based method in the V2 test, whereas the VT_2_ was only succesfully assessed in the NW test with the HFP_RSA_-based frequency-domain HRV method. Thus, one could encourage the use of the time-domain HRV methods in the aforementioned cases where they were proven successful, because they are cheap and fairly simple, although the applicability of the proposed frequency-domain analysis methods for practical settings are questioned due to being more complex, and hence, knowledge- and time-demanding.

We would propose to single out specific XC skiing protocols, using the DS technique to determine HRVT_1_ and the NW test for the assessment of HRVT_2_, rather than using all five techniques. Moreover, its applicability is yet to be evaluated in training routines. In any case, further research is needed to prove the validity and reliability of the proposed methods for the assessment of HRVTs in training routines, especially because there seem to be quite wide agreement interval for individual purposes. Further research is also needed for finding alternative HRV-based methods that will successfully assess the VTs in the cases where the present study failed. We suggest the use of nonstop incremental treadmill test protocols, with breath-by-breath analysis methods and time varying frequency-domain analysis of HRV for the assessment of the VT_2_. This would be even more time-demanding than the non-time varying frequency domain method used in this study, but it has already proven to be more efficient in ski-mountaineering [13, 14].

## Abbreviations

BF & PF: Breathing and poling frequencies
DP, DS, NW, V1 & V2: Five different cross-country (XC) skiing related techniques: double poling, diagonal striding, Nordic walking, and V1 and V2 skating
fHF &pfHF: Frequencies in HF range corresponding to BF and PF
HF & HFP: High frequency and HF power
HFP_LOC_& HFP_RSA_−LOC: and RSA-related components of HFP, respectively
HRVT_1-MSD_& HRVT_1-SD_: First HRVT determined from MSD and SD trends
HRVT_2-HFP_& HRVT_2-HFP-RSA_: Second HRVT determined from HFP and HFP_RSA_ trends
LF & LFP: Low frequency and LF Power
LOC: Cardiolocomotor coupling
MSD: Mean successive difference
RSA: Respiratory sinus arrhythmia

## References

[1] Meyer T, Lucía A, Earnest CP, Kindermann W. A conceptual Framework for Performance Diagnosis and Training Prescription from Submaximal Gas Exchange Parameters—Theory and Application. Int J Sports Med. 2005;26: 38–48. 10.1055/s-2004-830514 15702455

[2] Dourado VZ, Banov MC, Marino MC, de Souza VL, de O Antunes LC, McBurnie MA. A Simple Approach to Assess VT During a Field Walk Test. Int J Sports Med. 2010-,31: 698–703. 10.1055/s-0030-1255110 20617483

[3] Achten J, Jeukendrup AE. Heart Rate Monitoring. Applications and Limitations. Sports Medicine. 2003;33(7): 517–538. 12762827 10.2165/00007256-200333070-00004

[4] Freeman JV, Dewey FE, Hadley DM, Myers J, Froelicher VF. Autonomic Nervous System Interaction With the Cardiovascular System During Exercise. Progress in Cardiovascular Diseases. 2006;48(5): 342–362. 16627049 10.1016/j.pcad.2005.11.003

[5] Hartikainen JEK, Tahvanainen KUO, Kuusela TA. Chapter six: Short-Term Measurements of Heart Rate Variability In: Malik M, editor. Clinical Guide to Cardiac Autonomic Tests. Kluwer Academic Publishers;1998 pp.149–176.

[6] Task Force. Heart rate variability. Standards of measurement, physiological interpretation, and clinical use. European Heart Journal. 1996;17; 354–381. 8737210

[7] Anosov O, Patzak A, Kononovich Y, Persson PB. High-frequency oscillations of the heart rate during ramp load reflect the human anaerobic threshold. Eur J Appl Physiol. 2000;83: 388–394. 11138580 10.1007/s004210000302

[8] Blain G, Meste O, Bouchard T, Bermon S. Assessment of Ventilatory Thresholds during graded and maximal exercise test using time-varying analysis of respiratory sinus arrhythmia. Br J Sport Med. 2005;39: 448–452. 10.1136/bjsm.2004.014134 PMC1725254 15976169

[9] Cottin F, Lepretre P-M, Lopes P, Papelier Y, Médigue C, Billat V. Assessment of Ventilatory Thresholds from Heart Rate Variability in Well-Trained Subjects during Cycling. International Journal of Sports Medicine. 2006;27: 959–967. 17190003 10.1055/s-2006-923849

[10] Cottin F, Médigue C, Lopes P, Lepretre P-M, Heubert R, Billat V. Ventilatory Thresholds Assessment from Heart Rate Variability during an Incremental Exhaustive Running Test. International Journal of Sports Medicine. 2007;28: 287–294. 17024637 10.1055/s-2006-924355

[11] Karapetian GK, Engels HJ, Gretebeck RJ. Use of Heart Rate Variability to Estimate LT and VT. Int J Sports Med. 2008;29: 652–657. 10.1055/s-2007-989423 18213538

[12] Buchheit M, Solano R, Millet GM. Heart-Rate Deflection Point and the Second Heart-Rate Variability Threshold During Running Exercise in Trained Boys. Pediatric Exercise Science. 2007;19: 192–204. 17603142 10.1123/pes.19.2.192

[13] Mourot L, Fabre N, Savoldelli A, Schena F. Second Ventilatory threshold from heart rate variability: valid when the upper body is involved? Int J of Sports Physiology and Performance. 2014;9: 695–701. 10.1123/ijspp.2013-0286 24231307

[14] Cassirame J, Tordi N, Fabre N, Duc S, Durand F, Mourot L. Heart rate variability to assess Ventilatory thresholds in ski-mountaineering. 10.1080/17461391.2014.957729 25228474

[15] Di Michele R, Gatta G, Di Leo A, Cortesi M, Andina F, Tam E, et al Estimation of the Anaerobic Threshold from Heart Rate Variability in an Incremental Swimming Test. Journal of Strength and Conditioning Association. 2012;26(11): 3059–3066. 10.1519/JSC.0b013e318245bde1 22190158

[16] Fabre N, Perrey S, Arbez L, Rouillon J-D. Paced Breathing in Roller-Ski Skating: Effects of Metabolic Rate and Poling Forces. International Journal of Sports Physiology and Performance. 2007;2: 46–57. 19255454 10.1123/ijspp.2.1.46

[17] Holmberg HC, Calbet JAL. Insufficient ventilation as a cause of impaired pulmonary gas exchange during submaximal exercise. Respiratory Physiology and Neurobiology. 2007;157: 348–459. 17303477 10.1016/j.resp.2006.12.013

[18] Fabre N, Bortolan L, Pellegrini B, Zerbini L, Mourot L, Schena F. Anaerobic Threshold Assessment Through the Ventilatory Method During Roller-Ski Skating Testing: Right or Wrong? Journal of Strength and Conditioning Research. 2012;26(2): 381–387. 10.1519/JSC.0b013e3182260455 22228114

[19] Faria IE. Ventilatory response pattern of Nordic skiers during simulated poling. J Sports Sci. 1994;12:255–259. 8064972 10.1080/02640419408732171

[20] Holmberg HC, Lindinger S, Stöggl T, Eitzlmair E, Müller E. Biomechanical Analysis of Double Poling in Elite Cross-Country Skiers. Medicine & Science in Sports & Exercise. 2005: 807–818. 15870635 10.1249/01.mss.0000162615.47763.c8

[21] Durnin JVGA Rahaman MM. The assessment of the amount of fat in the human body from measurements of skinfold thickness. Br J Nutr. 1967;21: 544–550. 10.1079/bjn19670070 6052883

[22] Keskinen KL, Häkkinen K, Kallinen M, editors. Kuntotestauksen käsikirja. Finnish Society of Sport Sciences. 2007;156: 269.

[23] Westhoff M, Rühle KH, Greiwing A, Schomaker R, Eschenbacher H, Siepmann M, et al Positional paper of the German working group "cardiopulmonary exercise testing" to Ventilatory and metabolic (lactate) thresholds. Deutsche Medizinische Wochenschrift. 2013;138: 275–280. 10.1055/s-0032-1332843 23361352

[24] Saalasti S, Seppänen M, Kuusela A. Artefact Correction for Heartbeat Interval Data. Advanced Methods for Processing Bioelectrical Signals, ProBisi Meeting 2004 Available: Http://www.firstbeat.com/userData/firstbeat/download/saalasti_et_al_probisi_2004_congress.pdf.

[25] Hopkins WG, Marshall SW, Batterham AM, Hanin J. Progressive statistics for studies in sports medicine and exercise science. Med Sci Sports Exerc. 2009;41(1): 3–13. 10.1249/MSS.0b013e31818cb278 19092709

[26] Lamberts RP, Lambert MI. Day-to-Day Variation in Heart Rate at Different Levels of Submaximal Exertion: Implications for Monitoring Training. Journal of Strength and Conditioning Research. 2009;23(3): 1005–1010. 10.1519/JSC.0b013e3181a2dcdc 19387374

[27] Blain G, Meste O, Blain A, Bermon S. Time-frequency analysis of heart rate variability reveals Cardiolocomotor coupling during dynamic cycling exercise in humans. American Journal of Physiology—Heart and Circulatory Physiology. 2009;296: 1651–1659. 10.1152/ajpheart.00881.2008 19252094

